# Bullous Systemic Lupus Erythematosus as a Marker of Treatment-Refractory Systemic Disease: A Case Report

**DOI:** 10.7759/cureus.109261

**Published:** 2026-05-20

**Authors:** Alireza Izadian Bidgoli, Alberto Gomez Veliz, Joseph Mathew, Jordan De Guzman, Shabnam Yazdanpanah, Atharv Joshi

**Affiliations:** 1 Internal Medicine, American University of the Caribbean School of Medicine, Cupecoy, SXM; 2 Internal Medicine, Jackson Memorial Hospital, Miami, USA; 3 Pathology, Jackson Memorial Hospital/University of Miami, Miami, USA

**Keywords:** bullous systemic lupus erythematosus (bsle), cutaneous manifestations of systemic disease, systemic lupus erythema, systemic lupus erythematosus treatment, treatment resistant

## Abstract

Bullous systemic lupus erythematosus (BSLE) is a rare autoimmune blistering manifestation of systemic lupus erythematosus (SLE), typically regarded as a cutaneous variant with limited prognostic significance. However, its relationship to systemic disease activity and therapeutic response remains incompletely defined.

We present a 42-year-old male patient with known SLE and recently diagnosed BSLE, who developed progressive, widespread blistering lesions with mucosal involvement, dyspnea, and constitutional symptoms despite ongoing therapy. Physical examination revealed extensive tense bullae, erosions, and ulcerative plaques involving the extremities, face, and oral mucosa. Laboratory evaluation demonstrated positive autoimmune serologies consistent with SLE, while infectious and thromboembolic etiologies were excluded. Histopathology and direct immunofluorescence confirmed the diagnosis of BSLE. Despite treatment with systemic corticosteroids, hydroxychloroquine, dapsone, and rituximab, the patient experienced continued clinical deterioration requiring hospitalization and escalation of care.

This case underscores BSLE as more than a cutaneous manifestation, instead suggesting its role as a marker of aggressive, treatment refractory systemic disease. The persistence and progression of skin lesions despite appropriate immunosuppression paralleled ongoing systemic activity, indicating that refractory cutaneous findings should prompt reassessment of overall disease control rather than be dismissed as isolated dermatologic involvement. Early recognition of this pattern may support timely therapeutic escalation and closer multidisciplinary management, with potential to improve clinical outcomes.

## Introduction

Bullous systemic lupus erythematosus (BSLE) is a rare autoimmune blistering disorder that occurs in the setting of systemic lupus erythematosus (SLE), characterized by subepidermal blister formation and immunoglobulin deposition along the dermoepidermal junction [1]. Although BSLE is well described as a cutaneous manifestation of SLE, it is often considered a distinct dermatologic variant with relatively favorable response to conventional therapies, including corticosteroids and dapsone [2]. As a result, its broader clinical implications in reflecting systemic disease activity and treatment responsiveness may be underappreciated [1-3].

Emerging observations suggest that, in certain patients, BSLE may present with extensive mucocutaneous involvement and an atypical clinical course, raising the possibility that it reflects a more severe underlying immune dysregulation [1-3]. In such cases, the persistence or progression of blistering disease despite appropriate therapy may not represent isolated cutaneous resistance, but rather a manifestation of inadequate systemic disease control [1-3]. This distinction is clinically important, as it has implications for both risk stratification and therapeutic decision making [1,4].

Here, we present a case of severe, progressive BSLE in a patient with SLE whose disease course was characterized by worsening mucocutaneous and systemic symptoms despite standard and advanced immunosuppressive therapy. This case highlights the potential role of BSLE as a clinical indicator of aggressive and treatment refractory systemic disease and underscores the importance of recognizing disproportionate or nonresponsive cutaneous findings as a signal to reassess overall disease control. This case contributes to the limited literature describing BSLE as a potential clinical marker of systemic disease severity rather than an isolated cutaneous manifestation.

## Case presentation

Clinical presentation

A 42-year-old male patient with a known history of SLE and recently diagnosed with BSLE presented to the dermatology clinic for follow-up due to progressive disease despite ongoing therapy. He reported worsening blistering skin lesions, increasing fatigue, subjective fevers, dyspnea, and painful oral erosions that limited oral intake. At presentation, he appeared acutely ill with clear clinical deterioration compared to prior evaluations, prompting urgent referral to the emergency department for further workup and admission. His recent course was notable for hospitalization for a BSLE flare, during which he was initiated on rituximab induction therapy. 

Evaluation

**Figure 1:**
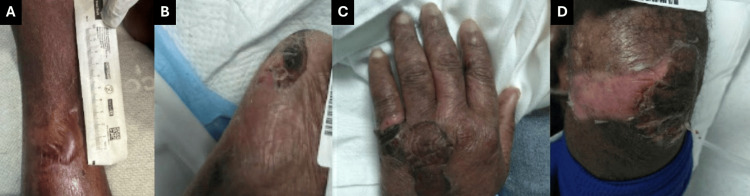
Cutaneous manifestations of BSLE involving the extremities (A) Lower extremity demonstrating a tense, newly formed bulla with surrounding erythema (B) Plantar foot showing an ulcerated, hyperpigmented lesion with overlying crust (C) Dorsal hand with a large, irregular hyperpigmented erosive plaque and adjacent digital involvement (D) Knee demonstrating ulcerated plaques with areas of necrosis and surrounding inflammation BSLE: Bullous systemic lupus erythematosus

**Figure 2:**
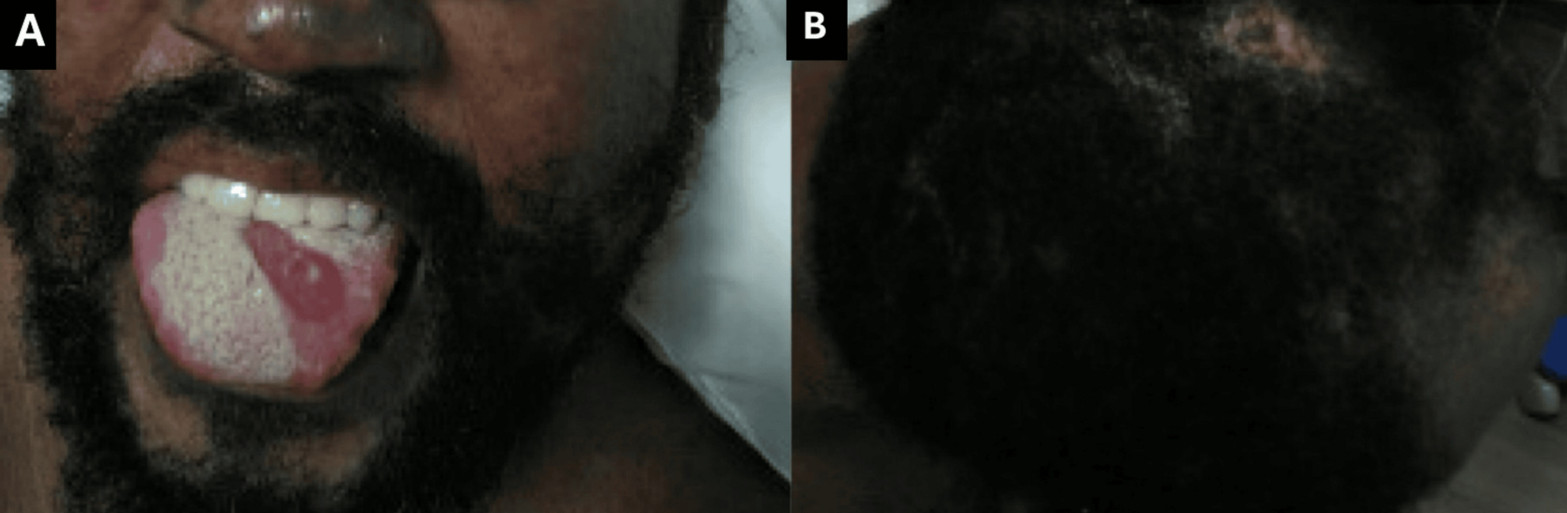
Facial and mucosal involvement of BSLE (A) Oral mucosa demonstrating a well-demarcated ulcerative lesion involving the anterior tongue with surrounding erythema (B) Scalp showing patchy non-scarring alopecia with overlying erythematous and eroded plaques BSLE: Bullous systemic lupus erythematosus

On examination, vital signs demonstrated tachycardia (heart rate >110 beats/min), borderline hypotension, and mild hypoxemia. The patient appeared ill but remained alert and oriented. The examination revealed widespread cutaneous involvement with multiple scattered bullae and erosions involving the extremities (Figure 1). Lesions ranged from intact tense bullae to ruptured lesions with overlying crust and exposed erosive surfaces. Several areas demonstrated ulceration with central crusting and surrounding hyperpigmentation. The dorsal hands and distal upper extremities showed irregularly shaped erosions with areas of necrotic appearing tissue and post inflammatory changes. The lower extremities, including the knees, exhibited larger ulcerated plaques with surrounding induration and adjacent smaller erosions (Figure 1). Additional findings included patchy alopecia of the scalp and cutaneous changes involving the face (Figure 2). Oral examination revealed multiple mucosal erosions involving the tongue and buccal surfaces (Figure 2). The remainder of the physical examination was unremarkable.

Laboratory evaluation demonstrated preserved metabolic and renal function despite significant clinical deterioration, although inflammatory and hematologic abnormalities were present, including elevated inflammatory markers, anemia, thrombocytosis, neutrophilia, and lymphopenia. Autoimmune serologies revealed elevated titers of antinuclear antibodies (ANAs) and extractable nuclear antigens. A detailed summary of laboratory findings is provided in Table 1.

**Table 1:** Summary of laboratory findings at presentation Reference ranges are provided for comparison. Abnormal values are categorized based on deviation from standard laboratory limits. ALT: Alanine aminotransferase; ANA: Antinuclear antibody; BUN: Blood urea nitrogen; CRP: C-reactive protein; dsDNA: Double-stranded DNA; eGFR: Estimated glomerular filtration rate; ESR: Erythrocyte sedimentation rate; RNP: Ribonucleoprotein; WBC: White blood cell count

Laboratory Parameter	Value at Presentation	Reference Range	Interpretation
General Chemistry/Renal Function			
Sodium (Na⁺)	135 mEq/L	135–145 mEq/L	Within normal limits
Potassium (K⁺)	4.9 mEq/L	3.5–5.0 mEq/L	Within normal limits
Bicarbonate (HCO_3_^-^)	26 mEq/L	22–28 mEq/L	Within normal limits
Anion Gap	5	8–16	Decreased
BUN	14 mg/dL	7–20 mg/dL	Within normal limits
Creatinine	0.83 mg/dL	0.6–1.3 mg/dL	Within normal limits
eGFR	>90 mL/min	>60 mL/min	Within normal limits
Albumin	3.4 g/dL	3.5–5.0 g/dL	Mildly decreased
ALT	61 U/L	7–56 U/L	Mildly elevated
Autoimmune Serologies			
ANA	Positive (1:1280)	Negative	Positive
Anti-Smith Antibody	Positive	Negative	Positive
Anti-RNP Antibody	Positive	Negative	Positive
Scl-70 Antibody	Positive	Negative	Positive
Anti-dsDNA	Negative	Negative	Within normal limits
Antiphospholipid Antibodies	Negative	Negative	Within normal limits
Inflammatory Markers/Hematology			
CRP	2.1 mg/dL	<1.0 mg/dL	Elevated
ESR	28 mm/hr	<20 mm/hr	Elevated
WBC	4.3 ×10³/µL	4.0–11.0 ×10³/µL	Within normal limits
Hemoglobin	11.1 g/dL	13.5–17.5 g/dL	Decreased
Hematocrit	33.2%	41–53%	Decreased
Platelet Count	419 ×10³/µL	150–400 ×10³/µL	Elevated
Neutrophils (%)	78.4%	40–70%	Elevated
Lymphocytes (%)	18.8%	20–40%	Mildly decreased
Absolute Lymphocyte Count	0.8 ×10³/µL	1.0–4.0 ×10³/µL	Decreased

Given the presence of dyspnea and tachycardia, chest imaging was obtained and demonstrated no acute cardiopulmonary abnormalities. CT angiography of the chest showed no evidence of pulmonary embolism. Additional findings included mild bilateral lower lobe interstitial changes, mosaic attenuation, and bilateral axillary lymphadenopathy. Electrocardiography demonstrated sinus tachycardia.

Treatment and tissue diagnosis

**Figure 3:**
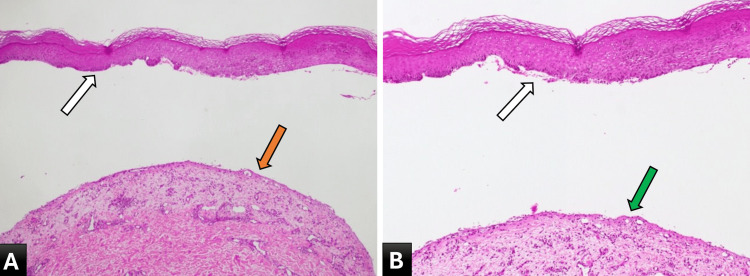
Histopathologic findings in BSLE Hematoxylin and eosin staining demonstrating a pauci-inflammatory subepidermal blister with separation at the dermoepidermal junction (white arrows). (A) Low-power view demonstrating subepidermal cleft formation with sparse inflammatory infiltrate within the blister cavity (orange arrow) (B) Additional section highlighting subepidermal blister formation and minimal inflammatory infiltrate along the blister base (green arrow) BSLE: Bullous systemic lupus erythematosus

**Figure 4:**
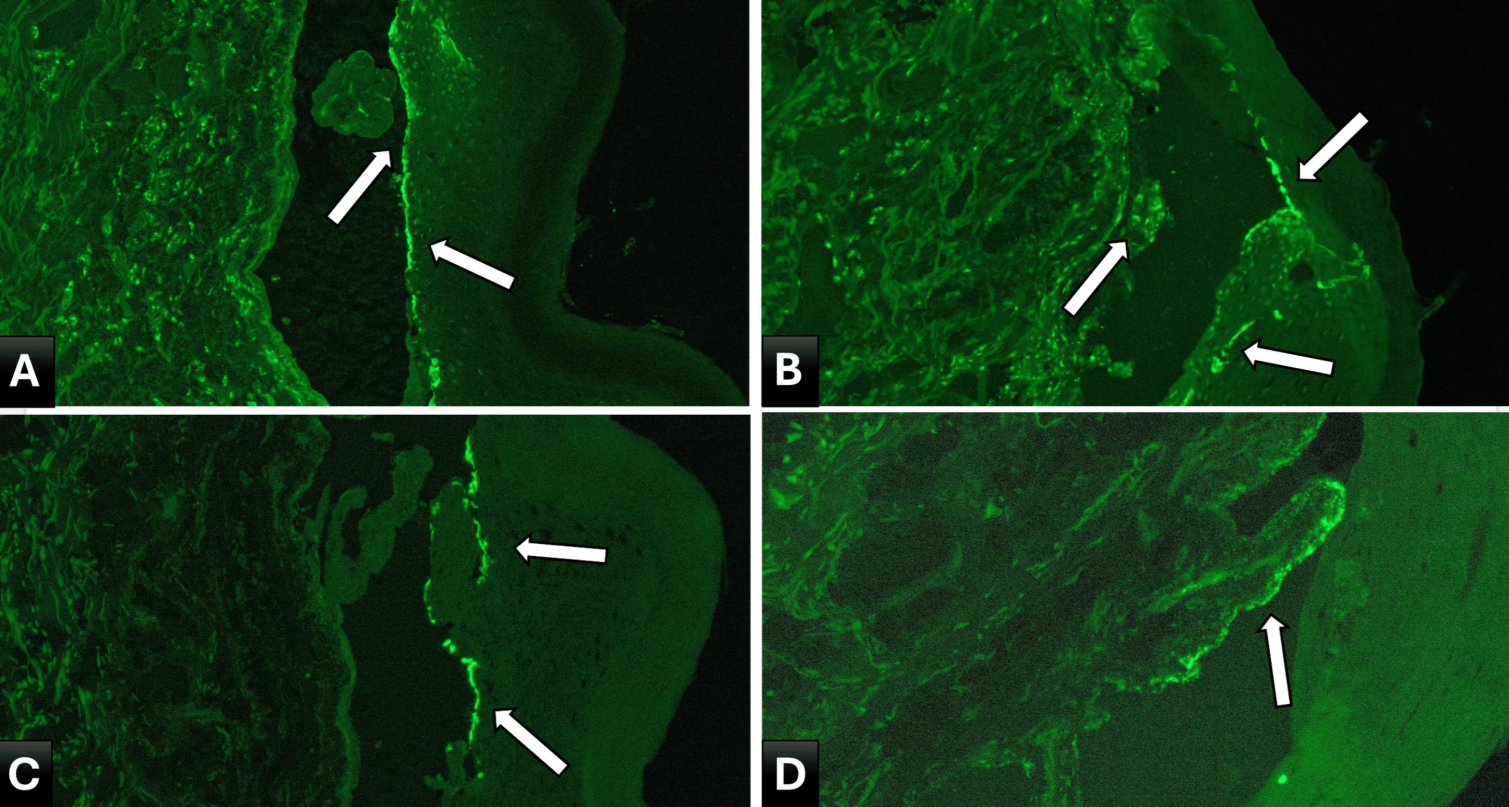
Direct immunofluorescence findings in BSLE Direct immunofluorescence microscopy demonstrating granular immune deposition along the dermoepidermal junction (white arrows). (A,B) Granular IgG deposition along the basement membrane zone (C,D) Granular C3 deposition along the basement membrane zone These findings support the diagnosis of BSLE in the appropriate clinicopathologic setting. BSLE: Bullous systemic lupus erythematosus; IgG: Immunoglobulin G

Prior skin biopsy demonstrated a pauci-inflammatory subepidermal blister with separation at the dermoepidermal junction and sparse inflammatory infiltrate within the blister cavity (Figure 3). Direct immunofluorescence showed granular deposition of immunoglobulin G (IgG) and C3 along the dermoepidermal junction, with fibrinogen positivity (Figure 4). Serologic testing for BP180, BP230, and desmoglein 1/3 antibodies was negative.

Prior to initiation of dapsone, glucose-6-phosphate dehydrogenase testing was performed and was within normal limits.

The patient was treated with hydroxychloroquine, systemic corticosteroids, dapsone, topical corticosteroids, topical antimicrobials, and rituximab induction therapy. At follow-up, due to worsening symptoms and examination findings, he was referred to the emergency department and admitted. During hospitalization, intravenous corticosteroids were initiated, and hydroxychloroquine and dapsone were continued. Topical corticosteroids were applied to cutaneous lesions, and supportive therapy was provided for oral mucosal involvement. Empiric intravenous antibiotics were initiated. A second rituximab infusion was planned during hospitalization.

Outcome

The patient was admitted for management of an uncontrolled BSLE flare with significant mucocutaneous involvement and concurrent respiratory symptoms concerning superimposed infection. Pulmonary embolism was excluded, and he remained hemodynamically stable following admission. Management focused on aggressive immunosuppression, supportive care, and treatment of suspected infection. Ongoing care involved multidisciplinary coordination with dermatology and internal medicine, with plans for continued immunomodulatory therapy and close outpatient follow-up.

## Discussion

Background (history, epidemiology, risk factors)

BSLE was first recognized as a distinct clinical entity in 1982 by Hall et al. [5]. It is a rare autoimmune blistering disease that occurs in the setting of SLE. Diagnostic criteria were subsequently proposed by Camisa and Sharma in 1983 and further refined in 1986, allowing clinicians to differentiate BSLE from other autoimmune blistering disorders [5,6]. These criteria include: (1) a diagnosis of SLE according to the American College of Rheumatology criteria; (2) vesicles and bullae arising on, but not limited to, sun-exposed skin; (3) histopathologic findings consistent with dermatitis herpetiformis; (4) negative or positive indirect immunofluorescence for circulating basement membrane zone antibodies using separated skin as substrate; and (5) direct immunofluorescence demonstrating linear or granular deposition of IgG and/or immunoglobulin M (IgM), and often immunoglobulin A (IgA), along the basement membrane zone [5,6]. When linear IgG deposition is present, immunoelectron microscopy may demonstrate immune reactants below the basal lamina [6].

An important clinical feature of BSLE is that lesions are not restricted to sun-exposed skin, as mucosal involvement may also occur [6]. Due to the rarity of the disease, most published data consist of isolated case reports or small case series [7]. This has limited the ability to accurately define the epidemiology and clinical spectrum of BSLE. A systematic review by de Risi-Pugliese et al. identified only 118 published cases of BSLE, underscoring the uncommon nature of this condition [7].

SLE affects an estimated 5 million people worldwide, with approximately 70% of lupus cases classified as SLE [8]. BSLE, however, represents a rare cutaneous manifestation of SLE, with reported prevalence estimates ranging from approximately 0.19% to 0.41% among patients with SLE [7,9]. The demographics of BSLE generally parallel those of SLE, occurring more commonly in females and individuals of African descent, although cases have been reported across diverse age groups, sexes, and ethnicities [7-9]. Clinically, BSLE may involve the trunk, extremities, face, and mucosal surfaces [6-8]. Early recognition and intervention are important to improve clinical outcomes [8]. Although the current literature contains increasing data regarding adult presentations of BSLE, reports in pediatric populations remain limited.

Dapsone remains the first-line therapy for BSLE and is often associated with rapid clinical improvement [8]. Additional therapies may include systemic corticosteroids and immunosuppressive agents such as methotrexate, cyclophosphamide, azathioprine, and mycophenolate mofetil, particularly in patients with extensive or refractory disease [8]. Because therapeutic response and disease severity may vary considerably, individualized treatment strategies are often required to optimize outcomes and minimize adverse effects.

Physiology/pathophysiology 

BSLE is an autoimmune blistering disease mediated by B cell-derived autoantibodies directed against type VII collagen, with neutrophils serving as the primary effector cells [8]. The development of these autoantibodies occurs in patients with SLE, where loss of self tolerance, B-cell hyperactivity, and type I interferon mediated immune activation create an environment that favors the emergence of pathogenic autoantibodies, including those directed against type VII collagen [9]. Type VII collagen is a key structural component of anchoring fibrils that secure the dermis to the epidermis. These autoantibodies target the noncollagenous (NC1 and NC2) domains of type VII collagen at the dermoepidermal junction. The NC1 domain is particularly important for its interactions with other basement membrane components, including type IV collagen, laminin 332, and fibronectin. Additionally, reports of autoantibody reactivity against laminin 332, laminin 311, and bullous pemphigoid antigen 1 suggest a role for epitope spreading in disease progression [8,10].

A majority of cases demonstrate IgG deposition along the basement membrane zone, although other immunoglobulin classes, including IgA and IgM, have been described [9,10]. The binding of these autoantibodies activates the classical complement pathway, leading to C5a mediated neutrophil recruitment and subsequent release of proteolytic enzymes that disrupt the dermoepidermal junction, resulting in blister formation [9,10].

Comparative analysis with current literature (clinical presentation, diagnostic workup, management, outcome)

*Clinical Presentation* 

BSLE represents a rare manifestation of SLE, with reported prevalence ranging from approximately 0.19% to 0.41% among SLE patients [1,7,9]. It may arise in individuals with established SLE or, less commonly, serve as an initial manifestation of systemic disease [1,4]. While BSLE most frequently affects females, particularly those of African descent in early to adulthood, it has been described across diverse demographic groups [1,7,9].

Clinically, BSLE is characterized by the abrupt development of tense vesicles and bullae involving the trunk, extremities, face, and, less commonly, mucosal surfaces [1,7]. Mucosal involvement, as observed in our patient, is less frequently reported and may reflect more severe disease activity [7,9]. Additionally, prior reports have suggested that extensive or rapidly progressive blistering may correlate with heightened systemic immune activation rather than isolated cutaneous disease [1,4]. In contrast to the typically manageable cutaneous course described in the literature, our patient demonstrated widespread, progressive lesions accompanied by constitutional and respiratory symptoms, indicating a more aggressive clinical phenotype.

Diagnostic Workup 

The diagnosis of BSLE requires integration of clinical features with histopathologic and immunologic findings [1,10]. Histologically, BSLE is characterized by subepidermal blister formation, often with neutrophil predominance, while direct immunofluorescence typically demonstrates deposition of immunoglobulins, most commonly IgG, and complement (C3) along the basement membrane zone [1,10]. Autoantibodies directed against type VII collagen further support the diagnosis and help distinguish BSLE from other vesiculobullous disorders [1,10].

In this case, biopsy revealed a pauci-inflammatory subepidermal blister with granular IgG and C3 deposition along the dermoepidermal junction, consistent with previously reported findings [1,9,10]. The differential diagnosis includes bullous pemphigoid, epidermolysis bullosa acquisita, linear IgA bullous dermatosis, dermatitis herpetiformis, and pemphigus spectrum disorders [1,11]. Negative serologic testing for BP180, BP230, and desmoglein antibodies, in combination with clinicopathologic correlation, effectively excluded these entities [10,11]. These findings underscore the importance of a comprehensive diagnostic approach incorporating histopathology, immunofluorescence, and serologic evaluation to accurately distinguish BSLE from other blistering diseases [1,10,11].

Management

Dapsone remains the first line therapy for BSLE and is associated with rapid clinical improvement in the majority of reported cases [2,11]. However, adjunctive therapies, including systemic corticosteroids, hydroxychloroquine, and other immunosuppressive agents, are frequently required in patients with more extensive or refractory disease [2,4,11].

In cases of treatment resistance, escalation to advanced therapies such as intravenous immunoglobulin or biologic agents, including rituximab, has been described with variable outcomes [2,12]. The therapeutic approach in our patient, including corticosteroids, hydroxychloroquine, dapsone, and rituximab, aligns with current management strategies for severe or refractory BSLE [2,4,11,12].

Importantly, emerging literature suggests that persistent cutaneous disease despite appropriate therapy may reflect ongoing systemic immune dysregulation rather than isolated dermatologic resistance [4,9]. This concept is clinically significant, as it supports the need for systemic reassessment and therapeutic escalation when cutaneous manifestations fail to improve.

Clinical Outcome

Most reported cases of BSLE demonstrate favorable outcomes, with rapid resolution of lesions and minimal long term sequelae following appropriate therapy [7,11]. However, refractory cases have been documented and are often associated with persistent systemic disease activity [9,11].

In contrast to the generally favorable outcomes described in the literature, our patient exhibited a severe and treatment refractory course characterized by progressive mucocutaneous involvement and systemic symptoms despite escalation to biologic therapy. This deviation from expected response suggests the presence of a more aggressive disease phenotype [4,9].

Furthermore, prior studies have indicated that the severity and persistence of BSLE may correlate with inadequate control of underlying SLE [9,11]. The clinical trajectory observed in this case supports this association and reinforces the importance of recognizing refractory BSLE as a potential indicator of uncontrolled systemic disease requiring prompt reassessment of therapeutic strategy [4,9,11].

A structured comparison with existing literature is summarized in Table 2, highlighting key differences in clinical presentation, diagnostic features, management strategies, and outcomes. While prior studies consistently report favorable responses to standard therapies, our case demonstrated persistent disease activity despite escalation to biologic therapy, suggesting a distinct treatment refractory phenotype. These findings support the evolving perspective that BSLE may function as a clinical marker of systemic disease severity rather than an isolated cutaneous manifestation.

**Table 2:** Comparative summary of BSLE in current literature vs present case BSLE: Bullous systemic lupus erythematosus; IgA: Immunoglobulin A; SLE: Systemic lupus erythematosus

Domain	Published literature	Present case	Clinical implication
Epidemiology	BSLE is a rare blistering manifestation of SLE, reported in approximately 0.19%-0.41% of lupus cohorts, and is described more commonly in women and patients of African descent [1,7,9].	42-year-old male patient with known SLE and recently diagnosed BSLE	This case falls outside the most commonly described demographic pattern and broadens the clinical profile in which BSLE should be considered [1,7,9].
Timing relative to SLE	BSLE may arise in patients with established SLE or may occasionally serve as the presenting manifestation of lupus [1,4,7].	Occurred in a patient with pre-existing SLE	Consistent with previously reported disease patterns and supports continued vigilance for BSLE during the course of established lupus [1,4].
Cutaneous findings	BSLE typically presents with acute tense vesicles and bullae involving the trunk, extremities, and face [1,7,8].	Widespread bullae, erosions, ulceration, crusting, hyperpigmentation, and necrotic-appearing lesions involving the extremities and face	The extent and progression of cutaneous disease in this case were more severe than the typically favorable cutaneous course described in prior reports [4,7,9].
Mucosal involvement	Mucosal involvement is a recognized manifestation of BSLE and has been described in reported case series and reviews [6,7,9].	Painful oral erosions involving the tongue and buccal surfaces	Supports a more extensive mucocutaneous phenotype and may reflect heightened systemic disease activity [4,7,9].
Systemic features	BSLE is frequently discussed as a cutaneous manifestation of lupus, although reported cases may coexist with active systemic disease [1,4,9].	Fatigue, subjective fevers, dyspnea, tachycardia, and clinical deterioration prompting admission	In this case, worsening skin disease paralleled broader clinical decline rather than isolated skin-limited disease [4,9].
Histopathology	BSLE classically demonstrates a subepidermal blister, often with neutrophil-rich inflammation [1,5,10].	Pauci-inflammatory subepidermal blister	Although less inflammatory than the classic description, the biopsy remains within the clinicopathologic spectrum of BSLE when interpreted with direct immunofluorescence and lupus context [5,10].
Direct immunofluorescence/immunopathology	Immune deposition along the basement membrane zone, often with IgG and C3, is characteristic of BSLE [5,6,10,11].	Granular IgG and C3 deposition along the dermoepidermal junction with fibrinogen positivity	The immunopathology is strongly supportive of BSLE in the appropriate clinicopathologic setting [5,6,10].
Exclusion of mimickers	Differential diagnosis includes bullous pemphigoid, epidermolysis bullosa acquisita, linear IgA bullous dermatosis, dermatitis herpetiformis, and pemphigus-spectrum disorders [1,10,11].	BP180, BP230, and desmoglein 1/3 antibodies were negative	The diagnostic workup appropriately helped exclude major competing autoimmune blistering disorders [10,11].
Standard therapy	Dapsone is widely described as first-line therapy and often produces rapid improvement; corticosteroids and systemic immunosuppressive agents are commonly added in severe disease [2,8,11].	Treated with dapsone, systemic corticosteroids, hydroxychloroquine, topical corticosteroids, and rituximab	Management was consistent with previously published escalation strategies for severe or refractory BSLE [2,4,11,12].
Response to treatment	Many reported patients improve with dapsone-based therapy, although refractory cases have also been documented [2,11,12].	Ongoing progression despite corticosteroids, dapsone, hydroxychloroquine, and rituximab	This case fits the uncommon refractory subgroup rather than the more typical treatment-responsive pattern [4,9,11].
Outcome	Reported outcomes are often favorable, with remission or substantial improvement following appropriate therapy [7,11].	Required hospitalization for uncontrolled flare with significant mucocutaneous involvement and ongoing need for escalation of care	Highlights a more aggressive clinical course than is typically emphasized in the literature [4,9,11].
Overall interpretation	BSLE is traditionally framed as a rare autoimmune blistering manifestation of lupus that is diagnosable through combined clinical, histologic, and immunologic findings and is often treatment-responsive [1,5,10,11].	Severe, progressive, treatment-refractory BSLE with systemic symptoms	Supports the central argument that refractory BSLE may function as a marker of inadequate systemic disease control rather than a purely cutaneous process [4,9,11].

What we learned from this case 

This case highlights the diagnostic and therapeutic challenges associated with autoimmune subepidermal blistering disease in patients with SLE. Although the patient’s clinical presentation, lupus-associated serologies, histopathologic findings, and direct immunofluorescence favored BSLE, overlap with epidermolysis bullosa acquisita remains an important diagnostic consideration given the shared immunopathologic features between these entities and the absence of specialized confirmatory testing such as salt-split skin indirect immunofluorescence or type VII collagen immunoblotting.

An important clinical insight from this case is that severe or treatment-refractory vesiculobullous disease in patients with lupus may reflect ongoing systemic immune dysregulation rather than isolated cutaneous involvement alone. Despite treatment with corticosteroids, dapsone, hydroxychloroquine, and rituximab, the patient demonstrated continued mucocutaneous progression and systemic symptoms requiring hospitalization and escalation of care. This clinical trajectory underscores the importance of reassessing overall disease activity in patients who fail to respond to standard therapies and supports the need for close multidisciplinary management in complex autoimmune blistering presentations.

## Conclusions

This case describes a patient with SLE presenting with a severe autoimmune subepidermal blistering disorder characterized by progressive mucocutaneous involvement and systemic symptoms despite immunosuppressive therapy. The clinical presentation, lupus-associated serologies, histopathologic findings, and direct immunofluorescence favored BSLE within the clinicopathologic spectrum of lupus-associated vesiculobullous disease. However, overlap with epidermolysis bullosa acquisita remains an important diagnostic consideration given the shared immunopathologic features between these entities and the absence of specialized confirmatory studies such as salt-split skin indirect immunofluorescence, immunoelectron microscopy, or type VII collagen-specific testing.

This case also highlights the potential association between severe or treatment-refractory vesiculobullous disease and ongoing systemic immune dysregulation in patients with lupus. Persistent mucocutaneous progression despite escalation of therapy should prompt reassessment of overall disease activity and consideration of multidisciplinary management and additional diagnostic evaluation when overlap autoimmune blistering disorders remain within the differential diagnosis. Further investigation is needed to better define the relationship between bullous lupus phenotypes, epidermolysis bullosa acquisita overlap syndromes, and systemic disease severity.
